# Cerebrospinal fluid tau levels are associated with abnormal neuronal plasticity markers in Alzheimer’s disease

**DOI:** 10.1186/s13024-022-00521-3

**Published:** 2022-03-28

**Authors:** Pieter Jelle Visser, Lianne M. Reus, Johan Gobom, Iris Jansen, Ellen Dicks, Sven J. van der Lee, Magda Tsolaki, Frans R. J. Verhey, Julius Popp, Pablo Martinez-Lage, Rik Vandenberghe, Alberto Lleó, José Luís Molinuevo, Sebastiaan Engelborghs, Yvonne Freund-Levi, Lutz Froelich, Kristel Sleegers, Valerija Dobricic, Simon Lovestone, Johannes Streffer, Stephanie J. B. Vos, Isabelle Bos, ADNI, August B. Smit, Kaj Blennow, Philip Scheltens, Charlotte E. Teunissen, Lars Bertram, Henrik Zetterberg, Betty M. Tijms, August B. Smit, Kaj Blennow, Philip Scheltens, Charlotte E. Teunissen, Lars Bertram, Henrik Zetterberg, Betty M. Tijms

**Affiliations:** 1 Alzheimer Center Amsterdam, Department of Neurology, Amsterdam Neuroscience, Vrije Universiteit Amsterdam, PO Box 7057 1007, MB Amsterdam, The Netherlands; 2 Alzheimer Center Limburg, School for Mental Health and Neuroscience, Maastricht University, PO Box 616, 6200 MD Maastricht, The Netherlands; 3 Department of Neurobiology, Care Sciences and Society, Division of Neurogeriatrics, Karolinska Institutet, Stockholm, Sweden; 4 Clinical Neurochemistry Laboratory, Sahlgrenska University Hospital, Mölndal, Sweden; 5 Department of Psychiatry and Neurochemistry, Institute of Neuroscience and Physiology, Sahlgrenska Academy at the University of Gothenburg, Mölndal, Sweden; 6 Department of Complex Trait Genetics, Center for Neurogenomics and Cognitive Research, Amsterdam Neuroscience, VU University, Amsterdam, the Netherlands; 7 Section Genomics of Neurodegenerative Diseases and Aging, Department of Clinical Genetics, Vrije Universiteit Amsterdam, Amsterdam UMC, Amsterdam, the Netherlands; 8 1St Department of Neurology, AHEPA University Hospital, Thessaloniki, Makedonia Greece; 9 Old Age Psychiatry, University Hospital Lausanne, Lausanne, Switzerland; 10 Department of Geriatric Psychiatry, University Hospital of Psychiatry and University of Zürich, Zürich, Switzerland; 11 Fundación CITA-Alzhéimer Fundazioa, San Sebastian, Spain; 12 Neurology Service, University Hospitals Leuven, Leuven, Belgium; 13 Laboratory for Cognitive Neurology, Department of Neurosciences, KU Leuven, Leuven, Belgium; 14 IIB-Sant Pau, Hospital de La Santa Creu I Sant Pau, Universitat Autonoma de Barcelona, Barcelona, Spain; 15 Barcelonaβeta Brain Research Center (BBRC), Barcelona, Spain; 16 Alzheimer’s Disease Unit and Other Cognitive Disorders Unit, Hospital Clinic de Barcelona, Barcelona, Spain; 17 Reference Center for Biological Markers of Dementia (BIODEM), Institute Born-Bunge, University of Antwerp, Antwerp, Belgium; 18 Department of Neurology, UZ Brussel and Center for Neurosciences, Vrije Universiteit Brussel, Brussels, Belgium; 19 Department of Psychiatry at School of Medical Sciences, Örebro University, Örebro, Sweden; 20 Department of Geriatric Psychiatry, Zentralinstitut Für Seelische Gesundheit, University of Heidelberg, Mannheim, Germany; 21 Complex Genetics Group, VIB Center for Molecular Neurology, VIB, Antwerp, Belgium; 22 Department of Biomedical Sciences, University of Antwerp, Antwerp, Belgium; 23 Lübeck Interdisciplinary Platform for Genome Analytics, Institutes of Neurogenetics and Cardiogenetics, University of Lübeck, Lübeck, Germany; 24 Jansen UK, High Wycombe, UK; 25 AC Immune SA, Lausanne, Switzerland; 26 Department of Molecular and Cellular Neurobiology, Center for Neurogenomics and Cognitive Research, Amsterdam Neuroscience, Vrije Universiteit Amsterdam, Amsterdam, the Netherlands; 27 Neurochemistry Laboratory, Department of Clinical Chemistry, Amsterdam University Medical Centers (AUMC), Amsterdam Neuroscience, Netherlands; 28 Center for Lifespan Changes in Brain and Cognition, Dept. of Psychology, University of Oslo, Oslo, Norway; 29 Department of Neurodegenerative Disease, UCL Institute of Neurology, London, UK; 30 Dementia Research Institute at UCL, London, UK

**Keywords:** Alzheimer's disease, Molecular mechanisms, Biomarker discovery, Heterogeneity, Neuronal plasticity, Cerebrospinal fluid proteomics

## Abstract

**Background:**

Increased total tau (t-tau) in cerebrospinal fluid (CSF) is a key characteristic of Alzheimer’s disease (AD) and is considered to result from neurodegeneration. T-tau levels, however, can be increased in very early disease stages, when neurodegeneration is limited, and can be normal in advanced disease stages. This suggests that t-tau levels may be driven by other mechanisms as well. Because tau pathophysiology is emerging as treatment target for AD, we aimed to clarify molecular processes associated with CSF t-tau levels.

**Methods:**

We performed a proteomic, genomic, and imaging study in 1380 individuals with AD, in the preclinical, prodromal, and mild dementia stage, and 380 controls from the Alzheimer’s Disease Neuroimaging Initiative and EMIF-AD Multimodality Biomarker Discovery study.

**Results:**

We found that, relative to controls, AD individuals with increased t-tau had increased CSF concentrations of over 400 proteins enriched for neuronal plasticity processes. In contrast, AD individuals with normal t-tau had decreased levels of these plasticity proteins and showed increased concentrations of proteins indicative of blood–brain barrier and blood-CSF barrier dysfunction, relative to controls. The distinct proteomic profiles were already present in the preclinical AD stage and persisted in prodromal and dementia stages implying that they reflect disease traits rather than disease states. Dysregulated plasticity proteins were associated with SUZ12 and REST signaling, suggesting aberrant gene repression. GWAS analyses contrasting AD individuals with and without increased t-tau highlighted several genes involved in the regulation of gene expression. Targeted analyses of SNP rs9877502 in *GMNC*, associated with t-tau levels previously, correlated in individuals with AD with CSF concentrations of 591 plasticity associated proteins. The number of *APOE-e4* alleles, however, was not associated with the concentration of plasticity related proteins.

**Conclusions:**

CSF t-tau levels in AD are associated with altered levels of proteins involved in neuronal plasticity and blood–brain and blood-CSF barrier dysfunction. Future trials may need to stratify on CSF t-tau status, as AD individuals with increased t-tau and normal t-tau are likely to respond differently to treatment, given their opposite CSF proteomic profiles.

**Supplementary Information:**

The online version contains supplementary material available at 10.1186/s13024-022-00521-3.

## Background

The amyloid cascade hypothesis poses that Alzheimer’s disease (AD) starts with amyloid beta (Aβ) aggregation followed by tau pathology [1]. Increased total tau (t-tau) levels in cerebrospinal fluid (CSF) are supposed to be caused by axonal loss [2]. Still, 25% of the AD individuals with mild cognitive impairment (MCI) or dementia have normal t-tau levels despite axonal loss [3, 4]. Moreover, CSF t-tau is abnormally increased in 50% of preclinical AD individuals, when neurodegeneration is limited [5]. Furthermore, our study in cognitively normal monozygotic twins discordant for amyloid aggregation, suggested that CSF t-tau levels may rise even before amyloid aggregation reaches abnormal levels [6]. An alternative explanation for the increase in t-tau in AD may be increased gene expression. A SILK study found that elevated CSF t-tau levels in AD were associated with increased production rather than from spilling of intracellular tau by dying neurons [7]. A study with induced pluripotent stem cells (iPSC) showed that AD individuals had increased tau expression in neuronal progenitor cells compared to controls [8]. Increased t-tau levels may also result of increased neuronal activity, [9] or increased neuronal plasticity as t-tau has an high expression in brain regions with plastic potential [10]. As the drivers of increased tau levels in AD remain largely unknown, and because tau-related mechanisms are emerging as new treatment targets for AD, we aimed to clarify molecular processes associated with CSF tau dysregulation. To this end, we performed a proteomic, genomic and imaging study including 1380 individuals in the AD continuum, [11] defined by abnormal CSF Aβ_1-42_, spanning the clinical spectrum from preclinical AD to mild dementia, and 380 controls with normal cognition and normal CSF Aβ_1-42_ and t-tau from the Alzheimer’s Disease Neuroimaging Initiative (ADNI) and the European Medical Information Framework for Alzheimer’s disease (EMIF-AD) Multimodality Biomarker Discovery (MBD) study [2].

## Methods

### Study participants

We selected 961 individuals from ADNI (adni.loni.usc.edu) and 799 individuals from the EMIF-AD MBD study [12]. ADNI started in 2003 as a public–private collaboration under the supervision of Principle Investigator Michael W. Weiner, MD. The primary goal of ADNI is to study whether serial biological markers, and clinical and neuropsychological measures can be combined to measure progression of MCI and early AD and has enrolled 2850 individuals, see www.adni-info.org. The EMIF-AD MBD study aimed to identify markers for diagnosis and prognosis of predementia AD. It combined existing clinical data, samples and scans of 1218 individuals with normal cognition, MCI or mild dementia from prospective cohort studies [12].

### Group definition and staging

We selected individuals with AD pathology defined as abnormally decreased CSF amyloid beta 1–42 (Aβ_1-42_), and we subdivided this group into those with abnormally increased total tau (t-tau) and those with normal t-tau. Based on cognitive performance, AD individuals were classified in 3 clinical stages as preclinical AD (normal cognition), prodromal AD (MCI) and mild AD-type dementia according to study specific criteria [12, 13]. The control group consisted of individuals with normal cognition, and normal CSF Aβ_1-42_ and t-tau levels.

### Clinical assessment

Global cognition was assessed by the Mini-Mental State Examination (MMSE) [14] and ADAS-Cog 11-item version (ADNI) [15]. As a measure for memory function we used the delayed recall of the logical memory subscale II of the Wechsler Memory Scale (ADNI), [16] or center specific verbal word learning tasks (EMIF-AD MBD) [12]. We selected the Clinical Dementia Rating (CDR) scale sum of boxes score as a measure of disease severity [17].

### CSF analysis

CSF samples in EMIF-AD MBD were collected according to the BIOMARKAPD protocol, [12, 18] and in ADNI as described elsewhere [19].

#### EMIF-AD MBD

CSF Aβ_1-42_ and t-tau were measured locally with INNOTEST ELISA or INNOBIA AlzBio3 (Fujirebio, Ghent, Belgium). Cut-offs for Aβ_1-42_ and t-tau were cohort-specific in EMIF-AD MBD [12]. Aβ_1-42_ cut-offs were determined for each cohort using Gaussian mixture modelling [20]. We measured neurogranin, neurofilament-light, YKL-40, Aβ_38_ and Aβ_40_ by ELISA [2]. We performed untargeted mass spectrometry using tandem mass tag (TMT) with 10 + 1 plexing as previously described, using high-pH reverse phase HPLC for peptide prefractionation [20, 21]. Peptides were mapped to 2535 proteins using the software ProteomeDiscoverer v.2.2. (Thermo Scientific), using Mascot (MatrixScience) for protein identification (precursor Dm tolerance 15 ppm, fragment tolerance 0.05 Da, max missed cleavage sites 2), searching the human subset of the UniProtKB SwissProt database (www.uniprot.org). Percolator (MatrixScience) was used for scoring peptide specific matches, and a strict 1% false discovery rate (FDR) was set as threshold for identification. For reporter ion quantification the following settings were used: Integration tolerance 20 ppm; Integration Method Most Confident Centroid; normalize on the reference protein average. The median (IQR) CV for these proteins was 5.6 (3.8, 8.0).

#### ADNI

Aβ_1-42_ and t-tau were measured on the xMAP platform (Luminex Corp, Austin, TX). Cut-offs for Aβ_1-42_ and t-tau were used as published [19]. Changes in t-tau levels over time were assessed in ADNI using longitudinal samples measured within the same batch. YKL-40, neurogranin, APP beta, neurogranin, neurofilament light, alpha synuclein, HBB, CFH, sTREM2, VILIP-1, and BACE1 were measured by ELISA or related assays, 190 analytes were analyzed by the Human DiscoveryMAP panel (MAP-RBM) and 225 proteins were analyzed by targeted mass spectroscopy [22, 23]. We used the quality checked and finalised ‘Normalized Intensity’ data as described in https://adni.loni.usc.edu/wp-content/uploads/2012/01/2011Dec28-Biomarkers-Consortium-Data-Primer-FINAL1.pdf

Proteins and protein fragments (ADNI) values were standardized according the mean and standard deviation values of the control group and expressed as z-scores, with a score of 0 indicating the average concentration of the control group, z-scores > 0 higher concentrations, and z-scores < 0 lower concentrations than controls. For ADNI, we averaged peptides that mapped to the same protein into a composite protein score when they correlated with r > 0.5, and included them as single peptides otherwise [20]. When the same protein was measured by different platforms in ADNI, values were averaged if they correlated with r > 0.5 and otherwise we included them as separate proteins. From the EMIF-AD MBD TMT mass spectrometry analysis proteins were included when observed in at least 92 individuals (30% of EMIF-AD MBD sample). For related proteins that had identical values due to fragment a-specificity (e.g. ACTA1 and ACTA2) we randomly selected one protein for analysis.

### Proteomic annotation

#### Enrichment analysis

We included proteins for enrichment analyses that were increased or decreased at *p* < 0.05 relative to the contrast tested.

##### Biological process enrichment analyses

We performed pathway enrichment analyses for Gene Ontology biological processes (GO-BP) using the online Panther application and SynGo [24–27].

##### Transcription factor enrichment analyses

We performed transcription factor enrichment analysis using ChEA through Harmonizome through Enrichr [28–30].

#### Predominant cell-type of protein production

Based on the BRAIN RNASeq database (http://www.brainrnaseq.org), [31] we labelled proteins as being predominantly produced by a cell type when levels were higher than 40% of older individuals produced across cell types.

#### Other annotations

Proteins associated with high choroid plexus expression were defined based on the Allen Brain Atlas, [32] through Harmonizome [29] and ABAEnrichment analysis [33]. Blood–Brain barrier (BBB) associated proteins were selected from reference [34–36]; BACE1 substrates were selected from reference [37, 38]; Alpha secretase substrates were selected from reference [39, 40]; and gamma secretase substrates were selected from reference [41].

#### Proteomic process scores (PPS)

For illustration purposes we combined proteins from selected GO-BPs, and proteins associated with BBB dysfunction and BACE1 substrates into a PPS by averaging z-scores of individual proteins belonging to the process that differed between AD individuals with normal or increased t-tau in cross-sectional analysis in the total group.

### Neuroimaging analysis

We studied neuroimaging data only from ADNI, because this study collected longitudinal scans. As a measure of brain atrophy, we took cortical thickness data from 34 cortical areas from the longitudinal processing pipeline in Freesurfer version 4.3 for 1.5 T T1-weighted MRI scans, and v5.1 for 3 T T1-weighted MRI scans (http://adni.loni.usc.edu). As a measure of amyloid accumulation, we used region-specific SUVR values for florbetapir binding assessed by PET imaging in 34 cortical areas as parcellated by Freesurfer v4.5.0 [42]. As a measure of brain metabolism we analysed fluorodeoxyglucose (FDG)-PET scans, [43] and determined average glucose metabolism for brain areas standardized to the average uptake in the vermis and brain stem according to the Desikan-Killiany atlas (http://adni.loni.usc.edu/methods/pet-analysis-method/pet-analysis/).

### Genomic assessment

EMIF-AD MBD samples were genotyped using the Illumina Global Screening array (Illumina, Incl) and 936 samples passed post-experiment QC [44]. ADNI samples were genotyped using the Illumina 2.5-M or Illumina OmniQuad array [45].

#### Genotype imputation and quality control

Data processing and quality control was performed using GenomeStudio Software (v2.0.04, Illumina, Inc.), as described previously [44]. To identify ethnic outliers, a principal component analysis (PCA) of ancestry was performed based on 1000 Genomes clustering, phase 3 using PLINK [44]. Individuals of non-European descent and family relations up to second degree were excluded. After filtering, the ADNI genotype data included 747 individuals and EMIF-AD MBD 931 individuals.

SNPs were locally imputed using Minimac 3 to the Haplotype Reference Consortium reference panel [46]. To account for population structure, principal components (PC1-PC20) were computed on a subset of relatively uncorrelated (r2 < 0.2) single nucleotide polymorphisms (SNPs). For ADNI subjects, Apolipoprotein E (*APOE*) ε4 genotype was assessed with two SNPs; rs429358 the ‘ε4-allele’ and rs7412 the ‘ε2 allele’. *APOE* ε4 genotypes in EMIF-AD MBD were generated as described elsewhere [44].

#### GWAS and post-GWAS analyses

Genome-wide association study (GWAS) analyses on increased CSF t-tau in subjects with abnormal Aβ_1-42_ were performed separately in ADNI (abnormal Aβ_1-42_ and increased t-tau *n* = 246, abnormal Aβ_1-42_ and normal t-tau *n* = 238) and EMIF-AD MBD (abnormal Aβ_1-42_ and increased t-tau *n* = 294, abnormal Aβ_1-42_ and normal t-tau *n* = 155) using PLINK software (V1.90). We used a logistic regression model including PC1-PC3 and sex. Genome-wide significance was defined as p ≤ 5e-08. Meta-analysis on ADNI and EMIF-AD MBD GWAS summary statistics was performed using METAL [47]. MAGMA software was used to compute gene scores and geneset scores for biological pathways based on p-values of the meta-analysis summary statistics. We considered for further analysis gene scores based on at least 6 SNPs and geneset scores with information on at least 6 genes.

#### Polygenic and other genetic risk score analysis

Polygenic risk scores (PGRS) for AD were computed for each subject using PRSice (V2.3) [48]. PGRS were calculated by adding the sum of each allele weighted by the strength of its association with AD risk as calculated previously [49]. AD cases were defined as patients clinically diagnosed with AD-type dementia or individuals with a parental history of AD (i.e., AD-by-proxy) [49]. Clumping was performed prior to calculating PGRS, to remove SNPs that are in linkage disequilibrium (r2 > 0.1) within a slicing 1 M bp window. After clumping, PGRS were computed using various SNP inclusion thresholds. In order to explore how specific genetic alterations were associated with CSF proteomic profiles, we calculated for a selection of genesets based on MAGMA analysis (see results), gene-specific risk scores (GRS) including only SNPs located on the respective genes with clumping as described above and computation of GRS using various SNP inclusion thresholds. We used weights from one cohort to generate GRS in the other cohort. In order to reduce the number of tests, GRS scores were selected for SNP inclusion threshold that best differentiated within AD between abnormally increased and normal t-tau.

### Statistics

Our main outcomes are the biological process and transcription enrichment analyses for which we report FDR corrected *p*-values. For other analysis we report uncorrected *p*-values. Test statistics can be found in the supplemental tables. Group comparisons between AD subgroups and controls were performed using ANOVA correcting for age and gender. Change in cognition and imaging markers were assessed with linear mixed models including as main terms group, time, and the interaction group by time, and correcting for age and gender, and additionally for level of education for cognitive markers. We investigated within the AD individuals with normal t-tau and AD individuals with increased t-tau whether protein concentrations increased or decreased with increasing disease severity ranging from preclinical AD, prodromal AD, to mild AD-dementia with linear models. A protein was considered to decrease or increase with disease severity if the linear trend was statistically significant or if there was a difference in concentration between preclinical AD and prodromal AD, preclinical AD and mild AD-dementia, or between prodromal AD and mild AD-dementia and the trend analysis supported the change with severity. Associations between GRS scores and CSF protein levels were tested with linear models adjusted for age and sex.

## Results

Of the individuals with AD, 788 (57%) had increased CSF t-tau levels and 592 (43%) normal t-tau levels. Abnormal and normal t-tau groups showed generally similar demographics and baseline cognitive performance (Data S1a).

We first determined stability of t-tau levels over time in 499 ADNI participants who had repeated CSF sampling up to 7 years. CSF t-tau increased in the prodromal AD stage at similar rates for both t-tau groups, and this increase did not differ from the increase in t-tau in the control group (Figure S1a). At last follow-up, 20% of the AD individuals with normal t-tau at baseline developed abnormally increased t-tau at follow-up and these individuals had t-tau levels just below the cut-point at baseline (Figure S1b). Taken together, these findings suggest that CSF t-tau levels reflect a trait rather than a stage.

**Fig. 1:**
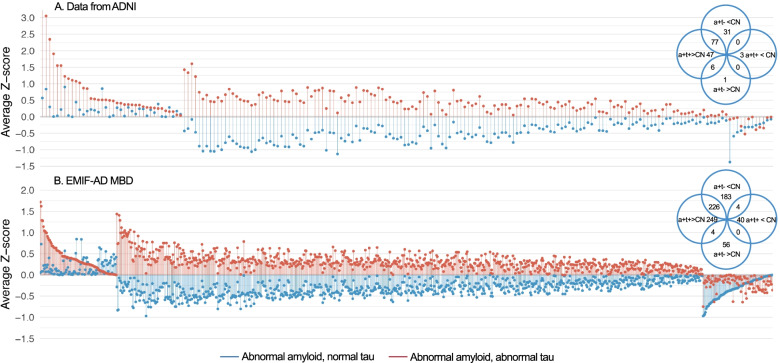
Protein concentrations relative to control group in individuals with AD according to t-tau status. Protein concentrations of individuals with abnormal Aβ_1-42_ and increased t-tau (a + t + , red) and abnormal Aβ_1-42_ and normal t-tau (a + t-, blue) in ADNI **A** and EMIF-AD MBD **B**. Concentrations are expressed as z-score relative to the control group (CN) that had normal cognition, normal Aβ_1-42_ and normal t-tau. Proteins are sorted according to change relative to control group. Shown are proteins that differed between individuals with AD with increased t-tau or AD individuals with normal tau from controls. Venn diagram shows number of proteins that differed

We then studied CSF proteome profiles for t-tau subgroups in 559 individuals with proteomic data (Data S1b). This included targeted proteomics of 248 proteins in ADNI and targeted and untargeted proteomics of 1458 proteins in EMIF-AD MBD (Data S2). Compared to controls, AD individuals with increased CSF t-tau had increased levels of 130 proteins in ADNI (52% of ADNI proteins measured) and of 477 proteins in EMIF-AD MBD (33% of EMIF-AD MBD proteins measured) and decreased levels of 2 proteins in ADNI (1% of proteins measured) and of 43 proteins in EMIF-AD MBD (3% of proteins measured) (Fig. 1, Data S2). AD individuals with normal CSF t-tau, showed an opposite pattern with increased levels of only 7 proteins in ADNI (3% of proteins measured) and of 60 proteins in EMIF-AD MBD (4% of proteins measured) and decreases in 107 proteins in ADNI (43% of proteins measured) and of 411 proteins in EMIF-AD MBD (28% of proteins measured). Around 50% of the proteins with increased levels in individuals with increased t-tau had decreased levels in individuals with normal t-tau. This indicates that t-tau groups show disruptions in the same molecular processes, but in opposite directions.

**Fig. 2:**
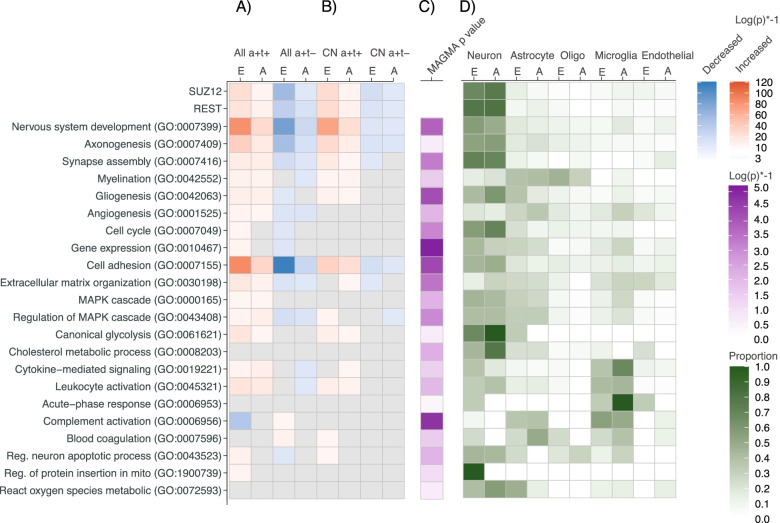
Association of selected GO biological processes and transcription factors with CSF proteins, MAGMA geneset score, and cell type. A,B GO-BP and SUZ12 and REST transcription factors enriched for proteins in AD individuals with increased t-tau (a + t +) or normal t-tau (a + t-) with increased (red) or decreased (blue) concentrations relative to controls in the total sample (A) or in the preclinical AD stage (B). *P*-values of all GO-BPs are listed in Data S3a. C *P*-value of association of GO-BP with abnormal t-tau in GWAS-based MAGMA genset analysis. *P*-values of all GO-BPs are listed in Data S4d. D Proportion of proteins with cell-type specific expression. A: ADNI cohort, E: EMIF-AD MBD cohort; A + t + : abnormal Aβ_1-42_ and increased t-tau; A + t-: abnormal Aβ_1-42_ and normal t-tau; CN A + t + : abnormal Aβ_1-42_ and increased t-tau in preclinical AD stage; CN A + t-: abnormal Aβ_1-42_ and normal t-tau in preclinical AD stage. Oligo = oligodendrocyte; endothelial = endothelial cell. (protein enrichment) and Data S4d (MAGMA analysis)

Enrichment analyses with GO-BP for CSF proteins increased in the AD group with abnormal t-tau relative to controls, showed in both ADNI and EMIF-AD MBD involvement of plasticity-related processes such as nervous system development, axonogenesis, synapse assembly, myelination, gliogenesis, angiogenesis, mitogen-activated protein kinase (MAPK) signaling, cell-cycle, gene expression and glycolysis (Fig. 2 selection of representative processes; Figure S2 shows synaptic processes; Data S3a, S3b show all enriched processes). Abnormality in each of these processes has been reported before in separate studies, [8, 50–55] and our findings now suggest that they are part of common plasticity response. We also observed involvement of cytokine signaling, leukocyte activation, oxidative stress, mitochondrial dysfunction, and apoptosis. Amyloid production was increased, reflected by increased levels of amyloid precursor protein (APP), Aβ_1-40_, and Aβ_1-38_, and increased levels and activity of BACE1, and increased concentrations in substrates of the main secretases involved in Aβ metabolism (i.e., BACE1, alpha and gamma secretase; annotated in Data S2, column DA-DC). Increased Aβ production is known to set off the amyloid cascade in autosomal dominant AD, [56] and our data suggest that increased amyloid production may also play a role in sporadic AD [57]. To identify potential drivers of increased protein levels we performed CHeA transcription factor-binding site enrichment analysis, which indicated SUZ12 (p-FDR corrected = 1.62E-11) and REST (p-FDR corrected = 1.04E-9) as most significantly enriched. SUZ12 and REST repress gene transcription through histone acetylation [58, 59]. Previous studies showed evidence of REST/SUZ12 de-repression in AD brain tissue, iPSC neurons from individuals with sporadic AD, and tangle bearing AD neurons [8, 60, 61]. Interestingly, of the proteins with increased gene expression in these previous studies that were measured in our CSF study, the majority showed increased CSF concentrations in AD individuals with increased t-tau: 4 of 4 (CALB1, NRXN3, SCN3B, SNAP25) proteins from reference [60]; 56 of 67 (84%) proteins from reference [8]; and 200 of 238 (84%) proteins from reference [61] (annotated in data S2, column DF-DG).

AD individuals with normal t-tau showed, relative to the control group, decreased levels of proteins associated with neuronal plasticity and regulation of MAPK signaling, with concomitant lower levels of APP, Aβ_1-40_ and Aβ_1-38_, and secretase substrates (Fig. 2, Figure S2; Data S3a, S3b for all enriched processes). Proteins with decreased concentrations in the AD group with normal t-tau, also converged on SUZ12 and REST transcription factors, suggesting that increased gene repression activity is driving this CSF proteomic profile. Furthermore, the AD group with normal t-tau showed 67 proteins with increased levels relative to controls. Twenty of these proteins were associated with blood–brain barrier (BBB) dysfunction (annotated in Data S2, column DD) and 25 proteins had a high expression in choroid plexus (ABAEnrichment minimum pFWER = 0.004), suggesting blood-CSF barrier (BCSFB) dysfunction (annotated in Data S2, column DE). Increased proteins were mainly seen in the EMIF-AD MBD cohort, as ADNI proteomics was targeted for brain specific proteins.

We continued analysis by comparing CSF protein concentrations between AD individuals with increased t-tau and normal t-tau directly. AD individuals with increased t-tau showed, relative to those with normal t-tau, an increase in the concentration of 167 proteins in ADNI (67% of ADNI proteins measured) and of 768 proteins in EMIF-AD MBD (53% of EMIF-AD MBD proteins measured) and a decrease in the concentration of 2 proteins in ADNI (1% of proteins measured) and of 136 proteins in EMIF-AD MBD (9% of proteins measured). These proteins were enriched for the same processes that differed for each group relative to controls (Figure S3, Data S3a). Analysis of cell-type specific proteins revealed that proteins of all major brain cell types differed in concentration between AD individuals with increased and normal t-tau and that plasticity proteins were typically neuron-specific (Fig. 2, Data S2).

**Fig. 3:**
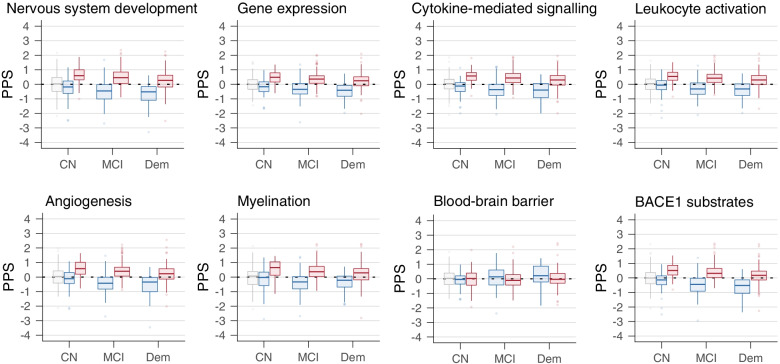
Association of protein process score (PPS) with disease severity. Data are based on cross-sectional analysis and combines data from ADNI and EMIF-AD MBD. In grey PPS of control group with normal cognition and normal CSF Aβ_1-42_ and t-tau; In red PPS of AD individuals with increased t-tau; In blue PPS of AD individuals with normal t-tau. CN = cognitively normal; MCI = mild cognitive impairment; Dem = dementia

We then investigated whether the opposite protein patterns could already be detected in the preclinical stage of AD. We found that relative to controls, preclinical AD individuals with increased t-tau had higher levels of plasticity proteins while preclinical AD individuals with normal t-tau showed lower plasticity protein levels. Preclinical AD individuals with normal t-tau also showed higher concentrations of proteins associated with barrier dysfunction (Table 1, Fig. 2b, Fig. 3, Data S2 column BK-BN, Data S3a for all enriched GO-BP). These findings indicate that both aberrant neuronal plasticity processes and barrier dysfunction are very early events in AD.

**Table 1:** Top 20 proteins that differed in preclinical AD from the control group according to CSF t-tau status

Contrast	Cohort	Number of proteins that differ	Top 20 proteins with largest effect size
**Preclinical AD with increased t-tau**			
Proteins with increased concentration relative to CN	EMIF-AD MBD	262	YWHAH, LAMP5, PCSK5, CHI3L1, SMOC1, ADCYAP1, SPP1, GDA, CRYM, TAGLN3, PLXNA2, PCDH8, HPRT1, CPD, GAP43, ENPP5, CAMK2A, PKM, MELTF, NAXE
	ADNI	93	NRGN, PKM, VSNL1, GOT1, ALDOA, SPP1, GOT2, HGF, NEO1, NCSTN, MOG, APLP2, SOD1, BACE1, APP, CHI3L1, FABP3, ENO2, NCAM2, SPON1
Proteins with decreased concentration relative to CN	EMIF-AD MBD	19	S100A6, ADAMDEC1, GPLD1, ANXA5, KNG1, SERPINA4, HSPA1A, IGHV4-30–2, CPB2, IGHV4-30–4, FLNA, IGHV3-30, PRAP1, IGLV3-10, HSPA6, ABI3BP, IGHV2-70D, HSPA7, PON1
	ADNI	2	GOLM1, LEP
**Preclinical AD with normal t-tau**			
Proteins with increased concentration relative to CN	EMIF-AD MBD	36	SLC39A12^b^, ADIPOQ^a^, ANGPTL7, LGI1^b^, CD9^b^, KRT24, IFI30^a^, TTR^b^, FOLR1^b^, NCMAP, SLC5A5^b^, SELPLG, ENPP2^b^, F5^b^, CTSA, KRT12, KRT9, SIAE^b^, COL15A1, KRT10
	ADNI	3	EDN1, APOE-e4, FGF4^b^
Proteins with decreased concentration relative to CN	EMIF-AD MBD	102	HS6ST1, CDH9, ASTN1, PLXNA1, APP, PMP2, STMN3, CDH8, PRKAR1A, GABARAPL2, GALNT6, PITHD1, GALNT1, CPM, MMP17, FAM19A2, CD99, FURIN, TAGLN, CDH12
	ADNI	24	abeta1-40, abeta1-38, ADGRL1, CADM3, NEO1, NPTX1, MCAM, CHGB, PCSK1, NEGR1, L1CAM, UBB, PTPRN, CACNA2D1, TIMP1, PAM, BTD, VEGFA, DAG1, NBL1

*T-tau* Total tau, *CN* Control group with normal cognition, normal Aβ_1-42_ and normal t-tau

^a^Protein associated with Blood Brain Barrier dysfunction

^b^Proteins with high expression in choroid plexus. Full list of proteins is provided in Data S2, column BK-BN

We next investigated how protein concentrations changed with increasing disease severity stages. In AD individuals with increased t-tau levels, the concentration of 59 (ADNI) to 129 proteins (EMIF-AD MBD) decreased with increasing disease severity and the concentration of 2 (ADNI) to 34 proteins (EMIF-AD MBD) increased. Proteins of which the concentration decreased with disease severity were enriched for neuronal plasticity and proteins of which the concentration increased were enriched for mitochondrial outer membrane permeabilization and complement activation (Fig. 3, Data S2, S3a (all enriched GO-BP), Figure S3). In AD individuals with normal t-tau, the concentration of 48 (ADNI) to 265 (EMIF-AD MBD) proteins decreased with increasing disease severity and the concentration of 10 proteins (ADNI) to 54 (EMIF-AD MBD) increased with disease severity (Fig. 3, Data S2, S3a (all enriched GO-BP), Figure S3). Proteins of which the concentration decreased with disease severity were enriched for neuronal plasticity and proteins of which the concentration increased proteins included BBB related proteins (*n* = 32) and proteins with increased expression in the choroid plexus (*n* = 14). Because the concentration of plasticity proteins decreased in both AD individuals with increased t-tau and normal t-tau, differences in plasticity proteins between the groups that were present in the preclinical AD stage persisted in the prodromal and dementia stage (Fig. 3). The decrease in concentration of neuronal plasticity proteins with disease severity aligns with the observation of decreased gene expression of neuronal plasticity proteins with increasing disease severity in post-mortem AD studies [57, 62].

**Fig. 4:**
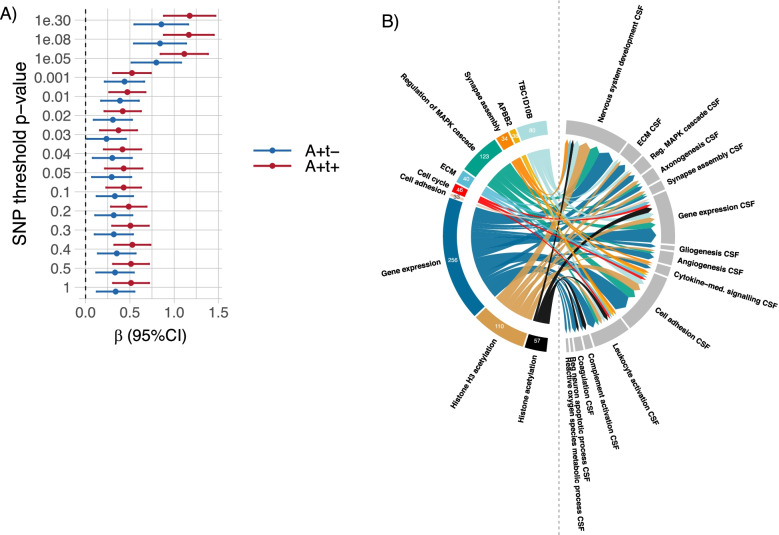
Genetic analysis. **A** PGRS across SNP thresholds SNP weights were based on [49]. Full data (including data after correction for APOE ε4 genotype and demographics, and separate for cohort and clinical stage) are shown in Supplemental Data S4a. a + t + : abnormal Aβ_1-42_ and increased t-tau (red); a + t-: abnormal Aβ_1_-_42_ and normal t-tau (blue). **B** Number of proteins associated with genetic risk score (GRS) and processes associated with these proteins. Left site shows number of proteins with a positive correlation with CSF t-tau GWAS-based GRS (coloured outer ring) and right site indicates to which GO-biological processes (GO-BP) these proteins below (GO-BP CSF, grey outer ring). Shown are the 10 GRS with the highest number of positive correlations with CSF proteins. The size of the arrows visualizes the number of correlations between GRS and protein concentration. For example, the GO-BP gene expression GRS showed a positive correlation with 256 proteins and these proteins were predominantly associated with nervous system development, gene expression and cell adhesion. Arrows to CSF GO-BP are shown in case the GRS correlated with at least 8 proteins from the CSF GO-BP. Supporting data in Data S5a. ECM = Extracellular matrix

Next, we investigated whether genetic factors were related to increased t-tau in AD individuals in the combined ADNI and EMIF-AD MBD cohorts (*n* = 1067). Presence of the *APOE* ε4 allele, the major genetic AD risk factor, was more common in AD individuals with increased t-tau compared to AD individuals with normal t-tau (66% vs 53% *p* < 0.001) and was in both AD groups more common than in controls (17%, *p* < 0.001). Compared to controls, AD individuals with increased t-tau and with normal t-tau had both higher AD PGRS across SNP inclusion thresholds (Fig. 4a, Data S4a) [49]. PGRS did not differ between AD individuals with increased t-tau and normal t-tau after stratification for clinical stage and correction for *APOE ε4* carriership and age (Data S4a), suggesting that these groups have a similar AD genetic risk architecture. Next, we performed an exploratory GWAS within AD individuals to identify other potential genetic markers associated with increased t-tau. We found tentative associations with SNPs in the *APBB2* gene (nominal *p*-value 1.63–2.92E-06, Data S4b). The top 3 genes associated with increased t-tau from gene-based analyses included, in addition to *APBB2*, *TBC1D10B* (nominal *p* = 2.9E-04), a Rab GTPA activating protein involved in MAPK signaling, and *LRP3* (nominal *p* = 3.36E-04), a low-density lipoprotein receptor protein (Data S4c). The top 3 GO-BP gene-sets associated with increased t-tau from MAGMA analysis were positive regulation of cellular process (self-contained *p* = 1.07E-04), protein K29-linked ubiquitination (self-contained *p* = 1.52E-04) and negative regulation of actin nucleation (self-contained *p* = 1.57E-04, Data S4d). Twenty-eight percent of GO-BPs enriched for CSF proteins that differed in concentration between AD t-tau groups, differed also in GO-BP gene-set score between the t-tau groups (Fig. 2; Data S3a). This suggests that interindividual differences in CSF t-tau concentrations may have partly a genetic basis. To investigate whether genetic markers influenced CSF protein concentrations, we correlated subject level GRS with subject level CSF protein concentrations in 188 individuals with abnormal Aβ_1-42_. We used the GRS of the top 3 genes, the GRS of the top 3 GO-BP and the GRS of 9 GO-BPs that differed between abnormal and normal t-tau groups in both CSF and MAGMA geneset analysis in Fig. 2. Given the association of CSF profiles with REST and SUZ12 we also selected the GRS of 2 GO-BPs associated with histone acetylation with the lowest self-contained *p*-value. For each of the GRS, we used weights from one cohort to generate a GRS in the other cohort. We then correlated the individual GRS of each gene or GO-BP with individual protein concentrations (Data S5a). A positive correlation means that a higher GRS is associated with a higher protein concentration. The largest number of positive correlations of GRS with CSF protein concentrations were observed for the GO-BP gene expression GRS (288 proteins), the GO-BP regulation of MAPK cascade GRS (146 proteins), and the GO-BP histone H3 acetylation GRS (127 proteins, Fig. 4b, left coloured part, Data S5a). The proteins that showed a positive correlation with these GRS were typically associated with nervous system development, gene expression, and cell adhesion (Fig. 4b, right grey part). These findings support the notion that the increase in plasticity-related processes in CSF has in part a genetic background. Moreover, we tested the association of SNP rs9877502 in *GMNC*, previously associated with CSF t-tau levels without stratification for amyloid aggregation, with tau status [44, 63, 64]. Consistent with previous data, the A-allele frequency at this site was more common in AD individuals with increased t-tau compared to those with normal t-tau (nominal *p*-value = 0.02). We then associated this SNP with CSF protein levels using additive models. 591 of 1705 proteins (35%) showed an increase in CSF level with increasing number of rs9877502 A-alleles (Data S5b). These proteins were enriched for neuronal plasticity related processes (Data S5c), and SUZ12 (p-FDR corrected = 5.27e-24) and REST (p-FDR corrected = 6.34e-18) signaling. 105 proteins (6%) showed a negative correlation with the number of rs9877502 A-alleles, including 36 proteins associated with BBB dysfunction and 36 immunoglobulins. In a smilar way, we associated the number of *APOE-e4* alleles with CSF protein levels in individuals with AD. Forty-five proteins showed an increase in CSF levels with increasing number of APOE-e4 alleles (Data S5b) and these proteins were enriched for GO-BP learning or memory (p-FDR corrected = 0.046). The concentration of 36 proteins showed a negative correlation with the number of *APOE-e4* alleles (Data S5b) and were not enriched for any GO-BP.

Finally, we studied the effect of t-tau on disease progression using longitudinal ADNI data.

**Fig. 5:**
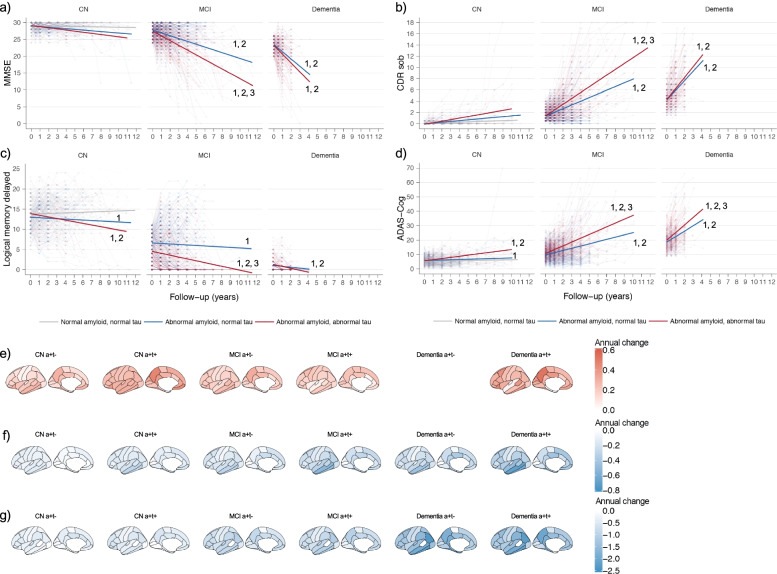
Longitudinal cognitive and imaging changes according to diagnostic group and clinical stage Upper images: change in cognitive markers. **A** MMSE, **B** CDR sum of boxes, **C** Logical memory, and **D** ADAS-Cog. Change of controls are shown in grey, of AD individuals with increased t-tau in red (a + t +), and of AD individuals with normal t-tau (a + t-) in blue. 1. Slope *p* < 0.05 different from control; 2. Slope *p* < 0.05 different from 0; 3. Slope *p* < 0.05 different between AD individuals with increased t-tau (a + t +) and AD individuals with normal t-tau (a + t-). Lower images: Annual rate of change in imaging markers. **E** amyloid aggregation, **F** cortical thickness and **G** FDG-PET glucose metabolism. PET amyloid data are not shown for AD individuals with dementia with normal t-tau because of low number of participants (*n* = 6). Data are shown in Data S6. Data are from ADNI only. CN = normal cognition; MCI = mild cognitive impairment; a + t +  = abnormal Aβ1-42 and increased t-tau; a + t- = abnormal Aβ1-42 and normal t-tau

Relative to controls, AD groups with increased t-tau and normal t-tau showed both faster memory decline and PET-amyloid accumulation in the preclinical stage and showed faster decline in all cognitive and imaging measures in the prodromal and dementia stage. Compared to AD individuals with normal t-tau, those with increased t-tau declined faster on cognitive tests and in glucose metabolism in the prodromal AD stage and showed a faster decline in cortical thickness in the prodromal and dementia stage (Fig. 5, Data S6).

## Discussion

Our main finding is that AD individuals with increased CSF t-tau levels showed increased concentrations of proteins associated with neuronal plasticity, while AD individuals with normal t-tau levels showed decreased concentrations of plasticity proteins. These opposite CSF proteomic profiles were already observed in the preclinical stage of AD, and persisted in prodromal and mild dementia stages, indicating that they reflect different disease traits rather than different disease states.

Results from our molecular process and transcription factor enrichment analysis suggested that the increased levels of neuroplasticity proteins in the AD group with increased t-tau could result from an increase in mitogenic MAPK signaling, or from a reduction in gene repression by REST and SUZ12. The de-repression of gene expression by REST/SUZ12 in AD has previously been reported in post-mortem AD studies and in iPSC neurons from individuals with sporadic AD [8, 60, 61]. Our finding that a subset of proteins coded by genes with increased expression in these previous studies had increased CSF concentrations in AD individuals with increased t-tau as well, further supports the role of REST/SUZ12 de-repression in AD. Both APP and tau are regulated by REST or SUZ12, and this may explain why the increases in levels of Aβ production markers and tau occur in tandem [6]. The increased concentration of neuronal plasticity proteins might reflect a compensatory mechanism triggered by tau-induced synaptic dysfunction such as impairment of presynaptic vesicle release, trafficking of glutamatergic receptors, and maturation of dendritic spines [65]. AD neuropathological studies suggested that such a plasticity response is likely pathological and results in aberrant synaptic sprouting (both at axons and at dendrites), disorganised capillairies and an abnormal cortical myelin architecture [51, 52, 66]. Increased expression of APP and tau may initiate a vicious cycle. APP and Aβ peptides can activate MAPK signaling, [53, 67, 68] which may further increase APP production[69, 70]. The APP intracellular domain (AICD) stimulates gene transcription, [71] and may increase expression of APP and BACE1 [72]. Abnormal tau may increase gene expression through depletion of H3K9me2, altered spatial open chromatin organization, or altered H3K9 acetylation [73, 74].

The group of AD individuals with normal t-tau was characterized by decreased concentrations of plasticity related proteins and increased concentrations of BBB and BCSFB permeability related proteins. The increase in barrier permeability may be caused by accumulation of Aβ in vessel walls and toxic effects of Aβ on cells that constitute these barriers [75, 76]. This can loosen tight junctions and increase paracellular transport [75, 77–79]. Impaired BBB and BCSFB function may lead to hypoplasticity through impairments in their physiological role in glucose transport, capillary perfusion, and neurogenesis, [79] and possibly also by increasing REST signaling, which can be triggered by ischemia [80]. The increase in concentrations of CSF proteins that are produced in the choroid plexus suggests BCSFB dysfunction. The choroid plexus is involved in the clearance of Aβ, [81] and the increase in concentration of proteins expressed in the choroid plexus could be a response to Aβ induced inflammation [82–84]. AD individuals with normal t-tau may have cerebral amyloid angiopathy (CAA), a condition in which Aβ is deposited in cerebral and leptomeningeal blood vessels [85]. CAA is common in individuals with AD and correlates with Aβ plaque deposition and neurofibrillary tangles [85]. CAA has been associated with normal levels of t-tau levels and a decrease in Aβ_40_ levels, which was also observed in our AD individuals with normal t-tau [86]. Although t-tau levels were within normal limits, this group showed a typical AD genetic pattern and a progressive disease course with increases in cognitive and AD biomarkers abnormalities, which supports the view that this is a subtype of AD, and not a different disease entity.

Our GWAS and GRS analyses suggested that levels of tau and neuroplasticity proteins partly depend on genes involved in gene expression, although no genome-wide significant signals were identified. *APBB2* showed the strongest association with CSF t-tau in these analyses. While markers in *APBB2* do not show strong evidence for association with AD risk in GWAS comparing controls with individuals with AD-dementia [49], the gene represents an interesting functional candidate as APBB2 binds AICD. Alternative splicing of *APBB2* increased the Aβ_42_/ Aβ_40_ ratio [87], and overexpression increased Aβ_1-40_, APP, and AICD levels and changed gene expression [68, 87, 88]. In addition, we found associations of rs9877502 in *GMNC* (previously associated with t-tau levels in this and other datasets) [44, 63, 64] with levels of 696 CSF proteins in AD individuals. *GMNC* is involved in neuronal plasticity and regulation of gene expression [89, 90]. We also found that GRS associated with t-tau were involved in neuronal plasticity and that these GRS showed an association with the concentration of plasticity-related proteins. On the contrary, the number of *APOEe4* alleles was not associated with the concentration of proteins involved in neuronal plasticity. The genetic pattern associated with t-tau levels in individuals with AD is thus different from the genetic processes associated with AD-type dementia, which include immunity, lipid metabolism, and intracellular trafficking [49]. This indicates that within the AD genetic risk architecture other genetic processes could influence AD phenotypes.

As we found a wide range of processes deregulated, it is possible that this may reflect an aspecific response to brain pathology. This seems, however, unlikely as the same set of proteins that was increased in AD individuals with increased t-tau was decreased in AD individuals with normal t-tau suggesting that these processes have a common denominator. In addition, we found that these proteins were enriched for SUZ12 and REST transcription factors, which are master regulators of neuronal plasticity. Moreover, abnormalities in nervous system development related processes, myelination, angiogenesis, MAPK signaling, cell-cycle, gene expression and glycolysis have all been reported in separate AD post-mortem, animal or cell studies [8, 50–55]. Still, some dysregulated processes may only be indirectly involved in plasticity. For example, increased levels of proteins associated with glycolysis could be a response to the high energy demand resulting from increased neuronal activity [91]. It is unlikely that the increased levels of plasticity proteins in AD individuals with increased t-tau simply reflect cell death as levels of plasticity-associated proteins were highest in the preclinical AD stage, when neurodegeneration is limited, and decreased with increasing disease severity.

The observation that increased t-tau levels are associated with an increase in concentration of plasticity proteins, aligns with observations that tau is associated with increased neuronal excitability in neurophysiological studies [92]. In an AD mice model it was shown that tau could be an enabler of neural network dysfunction caused by amyloid pathology as tau reduction reduced amyloid-related overexcitation [92, 93]. Tau may also have direct effects on excitability as overexpression of human tau in mice models increased delta/theta power and reduction of tau expression counteracted these changes [94]. Another study showed that nuclear translocation of tau led to increased expression of VgluT1, which is involved in glutamatergic synaptic transmission [95]. A clinical study showed that increased CSF t-tau levels were associated with long-term depression (LTD) after transcranial magnetic stimulation in the motor cortex [96]. As this study was performed in demented individuals, it is possible that LTD reflects synaptic failure. Future studies that combine electrophysiological measures with proteomic data in human beings are required to study this question in more detail.

We further found that AD individuals with increased t-tau also showed increased cytokine signaling and leukocyte activation. This activation could result from soluble tau oligomers, [97] although it is also possible that both increased t-tau levels and microglia activation result from amyloid pathology.

A strength of the study is the large sample size available for proteomic analysis and the use of two independent cohorts. Even though these cohorts performed proteomics in a different way (targeted proteomics in ADNI, non-targeted proteomics in EMIF-AD MBD) and the overlap in proteins measured in both studies was limited, enrichment analysis showed very similar results between the cohorts (Fig. 2), which supports the robustness of our findings. The consistency across cohorts was also present on the protein level. Of the 167 proteins measured in both cohorts, only 7 proteins (5%) showed a difference between the cohorts on both major outcomes (AD with normal t-tau vs control and AD with increased t-tau versus controls, Data S2). A limitation is that CSF proteomics was performed cross-sectionally and the differences between disease stages need to be confirmed in longitudinal studies. In addition, the association between change in CSF protein concentrations and biological processes needs to be further addressed in functional studies. The sample size for genetic analysis was small and larger samples are needed to confirm these results.

## Conclusions

CSF t-tau levels in AD are associated with altered levels of proteins involved in neuronal plasticity and blood–brain and blood-CSF barrier dysfunction. The association of increased CSF t-tau with neuronal plasticity protein levels provides support for the role of tau in neuronal plasticity and gene expression and implies that increased CSF t-tau levels may not simply reflect axonal loss [8, 73, 74]. It will be of critical importance to stratify future trials on t-tau status, as individuals with increased t-tau and normal t-tau are likely to respond differently to treatment, given their opposite proteomic profiles. The dosing of amyloid and tau antibodies may need to be tailored to t-tau levels, because of differences in BBB/BCSFB integrity between those with increased and normal t-tau. Treatments with drugs that target amyloid production, hyperexcitation, or hyperplasticity, such as bace inhibitors, histone modifiers and anti-epileptic drugs, could only be effective in AD individuals with increased t-tau in the predementia stage, when the CSF concentration of amyloid production markers and plasticity related proteins are highest. Retrospective analysis of previous trial data according to CSF t-tau status may lead to a better understanding why effects in these trials were absent or minimal and whether AD individuals with increased t-tau and normal t-tau have a different treatment response. Future studies on the mechanisms that lead to t-tau associated proteomic profiles, such as REST and SUZ12 signaling, will help to clarify AD pathophysiology and may eventually lead to novel drug targets.

## Supplementary Information

**Additional file 1:** **Data S1.** Participant characteristics. S1a: Characteristics of individuals with CSF Aβ1-42 and tau measurements available; S1b: Characteristics of individuals with CSF proteomic data. **Data S2.** Protein annotation and statistics of group comparisons of protein levels. Data S3a. Full list of GO biological processes associated with proteins that differ according to group and clinical stage. **Data S3b.** SynGO enriched synaptic cellular components and biological processes that differ according to group. **Data S4a.** Estimated marginal means of AD GWAS-based polygenic risk scores in controls, AD individuals with increased t-tau and AD individuals with normal t-tau. **Data S4b.** Top 1000 SNPS from GWAS on AD individuals with increased t-tau and normal t-tau in pooled ADNI and EMIF-AD MBD cohorts. **Data S4c.** Difference in MAGMA gene score between AD individuals with increased t-tau and normal t-tau based on t-tau GWAS in pooled ADNI and EMIF-AD MBD cohorts. **Data S4d.** Difference in GO biological process MAGMA geneset score between AD individuals with increased t-tau and normal t-tau based on t-tau GWAS in pooled ADNI and EMIF-AD MBD cohorts. **Data S5a.** Correlation between genetic risk score and CSF protein level in individuals with abnormal Aβ1-42. **Data S5b.** Association of the number of GMNC rs9877502-A risk alleles and number of APOE-e4 alleles with CSF protein concentrations in a linear model in individuals with AD. **Data S5c.** GO-BP processes enriched for proteins that have a positive or negative association with the number of rs9877502-A risk alleles in an additive model. **Data S6.** Annual change in imaging measures.

**Additional file 2:** **Figure S1.** Longitudinal change in CSF total tau. **Figure S2.** Enrichment of synaptic processes in individuals with AD according to t-tau status. **Figure S3.** Enriched GO biological processes and SUZ12 and REST transcription factors associated with proteins that differed between AD individuals with increased t-tau and normal t-tau and proteins that changed with disease severity.

## Data Availability

ADNI data can be downloaded from adni.loni.usc.edu. Raw proteomic data from EMIF-AD MBD has been deposited in the ProteomeXchange Consortium via the PRIDE partner repository with the dataset identifier 10.6019/PXD019910. Access to other EMIF-AD MBD data can be requested from the authors. Datasharing may be restricted by consent given by research participants within each contributing cohort and by European GDPR regulations, which currently exclude data sharing with a number of non-European countries. Statistical data can be found in the supplementary information files.

## Abbreviations

AD: Alzheimer's disease
ADNI: Alzheimer’s Disease Neuroimaging Initiative
AICD: APP intracellular domain
APOE: Apolipoprotein E
APP: Amyloid precursor protein
Aβ: Amyloid beta
BBB: Blood–Brain barrier
BCSFB: Blood-CSF barrier
CAA: Cerebral amyloid angiopathy
CDR: Clinical Dementia Rating
CN: Cognitively normal
CSF: Cerebrospinal fluid
ECM: Extracellular matrix
EMIF-AD: European Medical Information Framework for Alzheimers disease
FDG: Fluorodeoxyglucose
FDR: False discovery rate
GO-BP: Gene Ontology biological processes
GRS: Gene-specific risk scores
GWAS: Genome-wide association study
iPSC: Induced pluripotent stem cells
LD: Linkage disequilibrium
LTD: Long-term depression
MAPK: Mitogen-activated protein kinase
MBD: Multimodality Biomarker Discovery
MCI: Mild cognitive impairment
MMSE: Mini-Mental State Examination
PC1: Principal component
PCA: Principal component analysis
PET: Positron Emission Tomography
PGRS: Polygenic risk scores
PPS: Proteomic process scores
SNP: Single nucleotide polymorphisms
SUVR: Standardized uptake value
T-tau: Total tau
TMT: Tandem mass tag
