# Correction: Cerebrospinal fluid tau levels are associated with abnormal neuronal plasticity markers in Alzheimer’s disease

**DOI:** 10.1186/s13024-022-00540-0

**Published:** 2022-05-13

**Authors:** Pieter Jelle Visser, Lianne M. Reus, Johan Gobom, Iris Jansen, Ellen Dicks, Sven J. van der Lee, Magda Tsolaki, Frans R. J. Verhey, Julius Popp, Pablo Martinez-Lage, Rik Vandenberghe, Alberto Lleó, José Luís Molinuevo, Sebastiaan Engelborghs, Yvonne Freund-Levi, Lutz Froelich, Kristel Sleegers, Valerija Dobricic, Simon Lovestone, Johannes Streffer, Stephanie J. B. Vos, Isabelle Bos, ADNI, August B. Smit, Kaj Blennow, Philip Scheltens, Charlotte E. Teunissen, Lars Bertram, Henrik Zetterberg, Betty M. Tijms, August B. Smit, Kaj Blennow, Philip Scheltens, Charlotte E. Teunissen, Lars Bertram, Henrik Zetterberg, Betty M. Tijms

**Affiliations:** 1 Alzheimer Center Amsterdam, Department of Neurology, Amsterdam Neuroscience, Vrije Universiteit Amsterdam, PO Box 7057 1007, MB Amsterdam, The Netherlands; 2 Alzheimer Center Limburg, School for Mental Health and Neuroscience, Maastricht University, PO Box 616, 6200 MD Maastricht, The Netherlands; 3 Department of Neurobiology, Care Sciences and Society, Division of Neurogeriatrics, Karolinska Institutet, Stockholm, Sweden; 4 Clinical Neurochemistry Laboratory, Sahlgrenska University Hospital, Mölndal, Sweden; 5 Department of Psychiatry and Neurochemistry, Institute of Neuroscience and Physiology, Sahlgrenska Academy at the University of Gothenburg, Mölndal, Sweden; 6 Department of Complex Trait Genetics, Center for Neurogenomics and Cognitive Research, Amsterdam Neuroscience, VU University, Amsterdam, the Netherlands; 7 Section Genomics of Neurodegenerative Diseases and Aging, Department of Clinical Genetics, Vrije Universiteit Amsterdam, Amsterdam UMC, Amsterdam, the Netherlands; 8 1St Department of Neurology, AHEPA University Hospital, Thessaloniki, Makedonia Greece; 9 Old Age Psychiatry, University Hospital Lausanne, Lausanne, Switzerland; 10 Department of Geriatric Psychiatry, University Hospital of Psychiatry and University of Zürich, Zürich, Switzerland; 11 Fundación CITA-Alzhéimer Fundazioa, San Sebastian, Spain; 12 Neurology Service, University Hospitals Leuven, Leuven, Belgium; 13 Laboratory for Cognitive Neurology, Department of Neurosciences, KU Leuven, Leuven, Belgium; 14 IIB-Sant Pau, Hospital de La Santa Creu I Sant Pau, Universitat Autonoma de Barcelona, Barcelona, Spain; 15 Barcelonaβeta Brain Research Center (BBRC), Barcelona, Spain; 16 Alzheimer’s Disease Unit and Other Cognitive Disorders Unit, Hospital Clinic de Barcelona, Barcelona, Spain; 17 Reference Center for Biological Markers of Dementia (BIODEM), Institute Born-Bunge, University of Antwerp, Antwerp, Belgium; 18 Department of Neurology, UZ Brussel and Center for Neurosciences, Vrije Universiteit Brussel, Brussels, Belgium; 19 Department of Psychiatry at School of Medical Sciences, Örebro University, Örebro, Sweden; 20 Department of Geriatric Psychiatry, Zentralinstitut Für Seelische Gesundheit, University of Heidelberg, Mannheim, Germany; 21 Complex Genetics Group, VIB Center for Molecular Neurology, VIB, Antwerp, Belgium; 22 Department of Biomedical Sciences, University of Antwerp, Antwerp, Belgium; 23 Lübeck Interdisciplinary Platform for Genome Analytics, Institutes of Neurogenetics and Cardiogenetics, University of Lübeck, Lübeck, Germany; 24 Jansen UK, High Wycombe, UK; 25 AC Immune SA, Lausanne, Switzerland; 26 Department of Molecular and Cellular Neurobiology, Center for Neurogenomics and Cognitive Research, Amsterdam Neuroscience, Vrije Universiteit Amsterdam, Amsterdam, the Netherlands; 27 Neurochemistry Laboratory, Department of Clinical Chemistry, Amsterdam University Medical Centers (AUMC), Amsterdam Neuroscience, Amsterdam, Netherlands; 28 Center for Lifespan Changes in Brain and Cognition, Dept. of Psychology, University of Oslo, Oslo, Norway; 29 Department of Neurodegenerative Disease, UCL Institute of Neurology, London, UK; 30 Dementia Research Institute at UCL, London, UK

**Correction: Molecular Neurodegeneration 17, 1-16 (2022)**

**https://doi.org/10.1186/s13024-022-00521-3**

The original article [1] contained an error in co-author, Lars Bertram’s affiliation which has since been corrected.
