# Supplementary material for: Cerebrospinal fluid tau levels are associated with abnormal neuronal plasticity markers in Alzheimer’s disease
Source: Mol Neurodegener. 2022 Mar 28;17:27. doi: 10.1186/s13024-022-00521-3 (PMC8962234; doi:10.1186/s13024-022-00521-3)
Supplement: Supplementary file 2 — Additional file 2: Figure S1. Longitudinal change in CSF total tau. Figure S2. Enrichment of synaptic processes in individuals with AD according to t-tau status. Figure S3. Enriched GO biological processes and SUZ12 and REST transcription factors associated with proteins that differed between AD individuals with increased t-tau and normal t-tau and proteins that changed with disease severity. [file 13024_2022_521_MOESM2_ESM.docx]

ren

Supplemental Figures


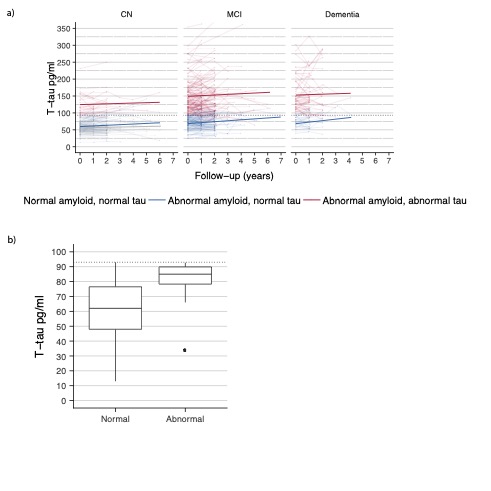


*

*

**Figure S1. Longitudinal change in CSF total tau**

A) Change in t-tau as function of group and stage. Grey=Control (n=98); Red=AD individuals with increased t-tau (CN n=27; MCI n=133, dementia n=57); Blue=AD individuals with normal t-tau (CN n=45; MCI n=110, dementia n=29). *Slope p<0.05 different from 0. CN= cognitively normal, MCI=mild cognitive impairment.

B) Baseline t-tau concentration of AD individuals with normal t-tau at baseline (n=184) according to t-tau status at last follow-up (normal tau: n=147 (80%); increased t-tau: n=37 (20%). Individuals with increased t-tau at follow-up typically had baseline t-tau levels just below the cut-point of abnormal t-tau (dotted line). Data are from ADNI.

Figure S2. Enrichment of synaptic processes in individuals with AD according to t-tau status

Upper row (A, B): synaptic processes enriched for proteins that showed higher levels in AD individuals with increased t-tau (a+t+) relative to controls (CN). Lower row (C, D): synaptic processes enriched for proteins that showed lower levels in AD individuals with normal t-tau (a+t-) relative to controls in EMIF-AD MBD (A, C) and ADNI (B, D). Note that, with a few exceptions, the same synaptic processes that are up-regulated in AD individuals with increased t-tau, are down-regulated in AD individuals with normal t-tau. Enrichment according to SynGO (www.syngoportal.org). Data are shown in Data S3b.

**Figure S3. GO biological processes and SUZ12 and REST transcription factors associated with proteins that differed between AD individuals with increased t-tau and normal t-tau and proteins that changed with disease severity.**

A) GO biological processes (selection, full list Data S3a) and SUZ12 and REST transcription factors enriched for proteins for which the concentration was higher (red) or lower (blue) in AD individuals with increased t-tau compared to AD individuals with normal t-tau.

B) GO biological processes (selection, full list sData 3a) and SUZ12 and REST transcription factors enriched for proteins that showed a decrease in concentration with increasing disease severity stage (blue) or an increase in concentration with increased disease severity stage (red). Data are shown for ADNI and EMIF-AD MBD separately. Heatmap shows p-value (log p-value*-1).
